# Suitable Environmental Ranges for Potential Coral Reef Habitats in the Tropical Ocean

**DOI:** 10.1371/journal.pone.0128831

**Published:** 2015-06-01

**Authors:** Yi Guan, Sönke Hohn, Agostino Merico

**Affiliations:** 1 Systems Ecology, Leibniz Center for Tropical Marine Ecology, Fahrenheitstraße 6, Bremen, Germany; 2 Jacobs University, Campus Ring 1, Bremen, Germany; Biodiversity Research Center, Academia Sinica, TAIWAN

## Abstract

Coral reefs are found within a limited range of environmental conditions or tolerance limits. Estimating these limits is a critical prerequisite for understanding the impacts of climate change on the biogeography of coral reefs. Here we used the diagnostic model ReefHab to determine the current environmental tolerance limits for coral reefs and the global distribution of potential coral reef habitats as a function of six factors: temperature, salinity, nitrate, phosphate, aragonite saturation state, and light. To determine these tolerance limits, we extracted maximum and minimum values of all environmental variables in corresponding locations where coral reefs are present. We found that the global, annually averaged tolerance limits for coral reefs are 21.7—29.6 °C for temperature, 28.7—40.4 psu for salinity, 4.51 μmol L^-1^ for nitrate, 0.63 μmol L^-1^ for phosphate, and 2.82 for aragonite saturation state. The averaged minimum light intensity in coral reefs is 450 μmol photons m^-2^ s^-1^. The global area of potential reef habitats calculated by the model is 330.5 × 10^3^ km^2^. Compared with previous studies, the tolerance limits for temperature, salinity, and nutrients have not changed much, whereas the minimum value of aragonite saturation in coral reef waters has decreased from 3.28 to 2.82. The potential reef habitat area calculated with ReefHab is about 121×10^3^ km^2^ larger than the area estimated from the charted reefs, suggesting that the growth potential of coral reefs is higher than currently observed.

## Introduction

Tropical coral reefs are among the most diverse ecosystems on Earth and have an enormous social and economic importance [1,2]. They account for less than 0.2% of the global ocean area [3,4] but provide habitats to about a quarter of all marine species [5]. They also provide goods and services to humans worth more than 170 billion US dollars per km^2^ each year [6]. The fitness of tropical corals depends on several environmental variables including temperature, salinity, nutrients, aragonite saturation state, and light. Like many other ecosystems, coral reefs are endangered by global environmental changes such as eutrophication, sea level rise, global warming, and ocean acidification [7,8].

In the last decades, many studies have documented the impacts of climate change on different coral reef ecosystems around the globe (see Dubinsky & Stambler, 2011, for an updated collection of studies) [9]. It is well established, for example, that rising sea temperature can cause widespread damage to reefs [10,11]. During the 20^th^ century, the global surface average temperature has increased by 0.74°C [12] and, concomitantly, temperature-driven bleaching events have increasingly been reported [13]. Besides an upper thermal tolerance limit, corals are also affected by a lower temperature threshold [14–17]. Other factors, such as salinity, nutrient concentrations, and aragonite saturation state can also affect coral growth [18–20]. Quantifying the responses of coral reefs to different environmental changes is therefore required to better understand their biogeography.

One of the first attempts to quantitatively predict the biogeography of coral reef global habitats on a global scale is represented by the works of Kleypas [21,22] with the use of the diagnostic model ReefHab. Using ReefHab in combination with environmental variables available up to the late ‘80s and early ‘90s, Kleypas [22] estimated the potential area of coral-reef habitat in tropical and subtropical regions. Since then, new marine environmental data are available. These new data can help to produce an updated view of potential reef habitats and can enable us to derive new tolerance limits for coral-reef habitats with respect to different environmental variables.

Here we use the ReefHab model in combination with the latest available environmental data and high-resolution bathymetry to predict the present day potential reef habitats for coral growth at the global scale. Our predictions are then discussed in the context of the actual observations of coral reef occurrences. Finally, by using ReefHab in an inverse mode, we determine new suitable environmental limits for coral reefs.

## Materials and Methods

### ReefHab model and environmental data

**Fig 1:**
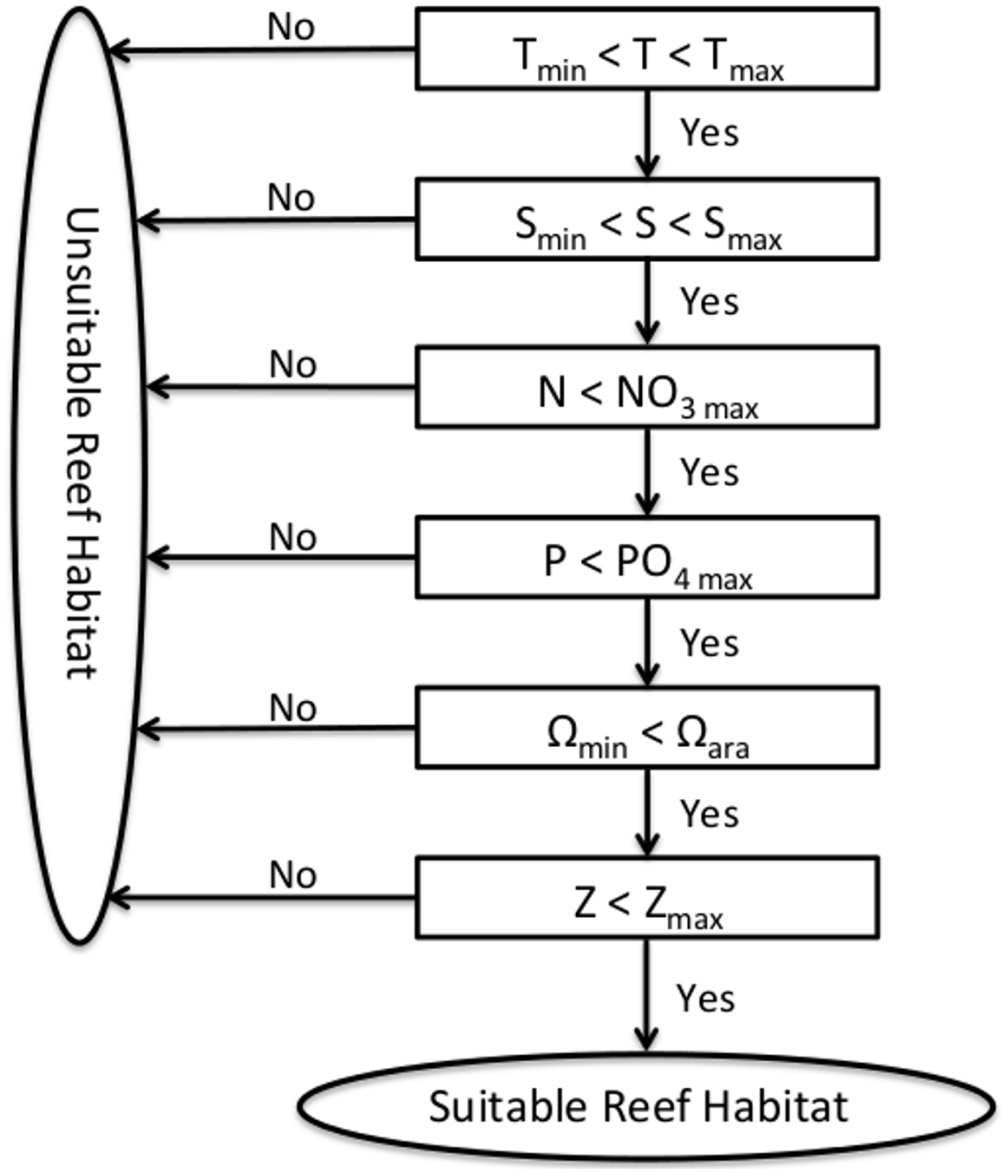
Flow chart of the ReefHab diagnostic model, modified from Kleypas (1997).

**Fig 2:**
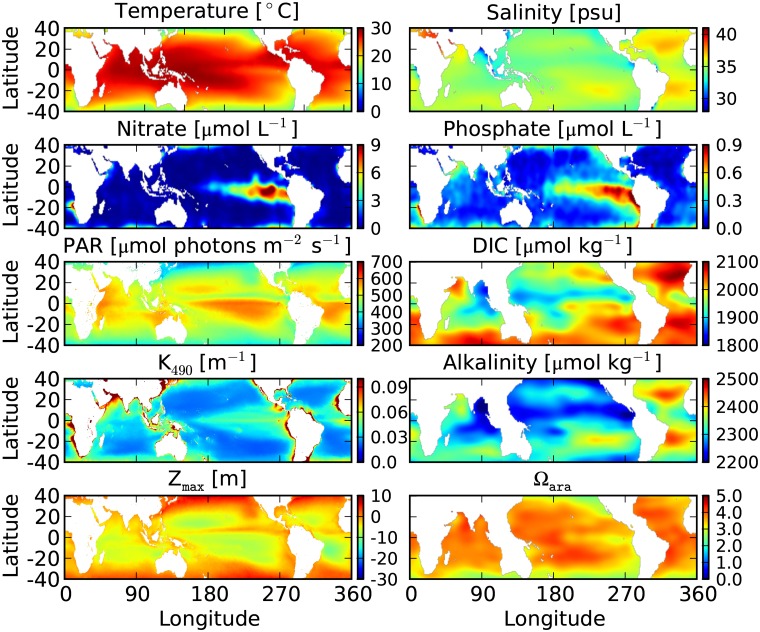
Environmental data used by the ReefHab model. Temperature, salinity, and nutrient data are taken from the World Ocean Atlas (WOA) 2009 and have a 1° × 1° spatial resolution. PAR and K_490_ are from SeaWiFS Level 3 data, have a 5’ × 5’ spatial resolution, and are used to calculate Z_max_ at *I*_min_ = 450 μmol photons m^-2^ s^-1^. Alkalinity and DIC are taken from GLODAP, have a 1° × 1° spatial resolution and are used to calculate Ω_ara_.

We use the diagnostic model ReefHab (Fig 1), which we coded in Python, to calculate the potential reef habitats for coral growth in the global ocean between 40° N and 40° S. The model uses climatological data of temperature (T), salinity (S), nitrate (${NO}_{3}^{-}$), and phosphate (${PO}_{4}^{3-}$) from the first 5 m water depth obtained from the World Ocean Atlas (WOA) 2009 [23–25] at a 1° × 1° spatial resolution (available at https://www.nodc.noaa.gov/OC5/WOA09/netcdf_data.html in netCDF format). Alkalinity and Dissolved Inorganic Carbon (DIC) at a 1° × 1° spatial resolution [26] (available at http://cdiac.ornl.gov/ftp/oceans/GLODAP_Gridded_Data/ in netCDF format) are used to calculate the aragonite saturation state (Ω_ara_) with the software CO2SYS [27], coded in Python. All these variables are shown in Fig 2.

The maximum depth of reef growth (Z_max_) is determined using the equation:

$${\text{Z}}_{\text{max}}=\frac{\text{ln}\left(I_{\text{min}}/\text{PAR}\right)}{{\text{K}}_{490}}$$ (1)

where *I*_min_ is the minimum light intensity necessary for reef growth (in μmol photons m^-2^ s^-1^), PAR (in μmol photons m^-2^ s^-1^) is the average photosynthetically available radiation at sea surface, and K_490_ (in m^-1^) is the attenuation coefficient of light at wavelength 490 nm. Both PAR and K_490_ are from SeaWiFS Level 3 data (available at http://oceancolor.gsfc.nasa.gov/cgi/l3) and have a spatial resolution of 5’ × 5’. The model calculates Z_max_ in each 5’ × 5’ grid cell. This information is then used in combination with the high-resolution (30” × 30”) bottom topography data from the General Bathymetric Chart of the Oceans (the GEBCO_08 Grid, version 2010, available at http://www.gebco.net in netCDF format) to check for the light criteria. The smallest 5’ × 5’ grid cell of PAR and K_490_ data is therefore subdivided into 100 cells of 30” × 30” resolution to match with bathymetry.

The model checks every 1° × 1° grid cell of the 360 × 80 matrix if temperature, salinity, nitrate, phosphate, and Ω_ara_ are within the specified ranges for potential reef habitat and every 30” × 30” grid cell of the 43200 × 9600 matrix if also the light condition is suitable. If all these variables are within the suitable ranges, the model produces a positive result in terms of suitable reef habitat at the given location. Otherwise, if any of these environmental variables is not in the suitable range, a negative result (i.e. unsuitable reef habitat) is generated (Fig 1). Ω_ara_ is not checked for in the Indonesian Sea and in the Caribbean because GLODAP does not contain DIC and alkalinity data in these regions. ReefHab predicts potential reef habitats at the same resolution as the topography dataset (30” × 30”) because water depth variations occur over small scales and exert a strong control over reef distribution. The spatial resolution of the environmental variables is much coarser (1° × 1°); however, these data do not vary considerably within their respective resolutions. The model results are finally presented on a 1° × 1° spatial resolution map and the percentage of potential reef habitat is calculated based on the percentage of positively evaluated 30” × 30” grid cells falling within a 1° × 1° grid cell.

Our results are compared with the works of Kleypas [22,28], which reproduced the potential reef habitat of the early ‘90s, by using temperature [29], salinity [30], nutrients [31], water depth [32], PAR [33], and K_490_ [34] with spatial resolutions of, respectively, 1° × 1°, 1° × 1°, 1° × 1°, 5’ × 5’, 2.5° × 2.5°, and 0.16° × 0.16°. The temporal resolution of temperature was weekly, all the other variables had monthly resolutions. Our study, however, predicts potential reef habitats based on the newest available environmental and topography data. In addition, we modified ReefHab by including a check on the aragonite saturation state.

### Reef location data

The model results (i.e. the potential reef habitats predicted with ReefHab) are qualitatively compared against charted reef observations of the Global Distribution of Coral Reefs 2010 [4,35,36] (available at http://data.unep-wcmc.org/datasets/13 as DBF data, which we transformed in HDF). These observations have been compiled from a variety of sources. Deep and cold water corals are not included in this study. The majority of the data, 85%, originates from the Millennuim Coral Reef Mapping Project and are mapped at a 30 m resolution. Of this large data fraction, only 35% has been validated [37]. The remaining 15% of the data were compiled from other sources, including the World Atlas of Coral Reefs [4]. Although this dataset has limitations, for example for some reef structure smaller than 30 m and in turbid areas, and despite the fact that only a small portion of it has been validated [37], it still represents the best and most used information available to date [38,39]. In this dataset, coral-reef areas are recorded as polygons. By overlaying these polygons on the available bathymetric profile (GEBCO_08) we created an "observed" reef habitat distribution on a 30” × 30” grid cell resolution. Every 30” × 30” grid cell that contains one or more points that constitute a coral-reef polygon is marked as observed reef habitat. For comparability with the coarse resolution environmental data, we calculated the percentage of observed reef habitats based on the number of 30” × 30” grid cells containing coral reefs within a 1° × 1° grid cell.

### Derivation of suitable environmental ranges for coral-reef habitats

To find the suitable environmental ranges for coral-reef habitats in today’s ocean, we used the model in an inverse mode, as explained in the following. We identified the values of annual temperature, salinity, nitrate, phosphate, and irradiance at each location (i.e. each grid cell) where observations showed the presence of reefs. We then considered the global maximum and minimum values of each environmental variable. These values represent the average environmental ranges for observed coral reefs and are later used with the model to predict the potential reef habitats, i.e. all those locations of the oceans, besides those already known from the observation, that can potentially host coral reefs.

The overlay of the observed reef locations with the GEBCO_08 bathymetry revealed inconsistencies between ocean depth and reef occurrence by showing the presence of coral reefs in waters deeper than 2000 m and up to 6000 m (see S1 Text and S1 Fig). Such inconsistencies remained even when using different bathymetry data (SRTM30, from ftp://topex.ucsd.edu/pub/srtm30_plus/). The calculation of the minimum irradiance (*I*_min_) required for coral growth (Eq 1), therefore, produced unrealistically low irradiance levels in locations supposedly associated with the presence of coral reefs but corresponding to very deep waters. In order to determine the most realistic value of *I*_min_ and hence circumvent such inconsistencies, we adopted a standard optimization technique. This consisted in systematically varying the value of *I*_min_ over a defined range to minimize the number of false negatives, while producing the most reasonable qualitative match between predicted potential reef habitat and actual reef distribution (see below for further details).

In order to analyse how model performance changes when using different tolerance limits, we run the ReefHab model with the most recent environmental datasets (WOA 2009) but in combination with the tolerance limits of Kleypas, hereafter K97 tolerance limits. In addition, we compare and discuss our results against the tolerance limits later suggested by Kleypas et al. [28], hereafter K99 tolerance limits.

Finally, our newly derived tolerance limits are determined on the basis of annual climatologies and do not take into account short-term (weekly, monthly, or seasonal) extremes. Although short-term disturbances can have lethal consequences for corals, it is the long-term (decadal) environmental condition that determines the presence/absence of coral reefs and that is relevant to our study. However, for comparability, we also calculated the tolerance limits on the same temporal scales (i.e. weekly and monthly) considered for deriving the K97 and K99 limits.

### Evaluation of model performance

The evaluation of the model performance consists of two major aspects: 1) the spatial pattern of the predicted potential reef habitats is compared with the observed coral-reef distribution on a 1° × 1° spatial resolution, and 2) the area of predicted potential reef habitats is compared with the area determined from observed reefs.

In order to compare the distribution pattern of predicted potential reef habitats with the observed coral reefs, we produced a 360 × 80 matrix of ones and zeros for, respectively, the presence (when the percentage of reef habitat is above 0) and absence of coral reefs (when the percentage of reef habitat equal 0). A similar matrix was produced for the observed coral-reef distribution. By subtracting the matrix of predicted potential reef habitats from the matrix of observed coral reefs, we generated a spatial distribution matrix with -1, 0, and +1. The value -1 represents a false positive (FP), i.e. the model predicts a suitable reef habitat in a grid cell where coral reefs are not observed. The value +1 represents a false negative (FN), i.e. the model does not predict a suitable reef habitat in a grid cell where reefs are actually observed. The value 0 reflects a match between model results and observed reefs and represents both a true positive (TP) and a true negative (TN).

To evaluate the response of the model to changes in *I*_min_, we used the Receiver Operating Characteristics (ROC) graph [40], by plotting the true positive rate vs. the false positive rate. The true positive rate (TPR) is the ratio between true positives and positives (P), i.e.: TPR = TP/P = TP/(TP + FN). The false positive rate (FPR) is the ratio between false positives and negatives (N), i.e.: FPR = FP/N = FP/(FP + TN). The data falling on the point TPR = 1 and FPR = 0 represent a perfect classification (i.e. a perfect model result). The distance to the perfect classification point can thus be used as a measure of the quality of the model results.

## Results

### Derivation of new tolerance limits

Table 1 summarises our results concerning the derivation of the new tolerance limits for the presence of coral reefs. We found that coral reefs are currently present in waters with annual mean temperature between 21.7°C and 29.6°C and with annual mean salinity between 28.7 psu and 40.4 psu. These values are not very different from those by K97. In contrast, the nitrate threshold above which no corals are found has increased from 2.0 μmol L^-1^ (K97) to 4.51 μmol L^-1^ (this study) and the phosphate threshold has increased from 0.2 μmol L^-1^ (K97) to 0.63 μmol L^-1^ (this study). Note, however, that the K97 tolerance limits were initially based on values quoted in the literature and subsequently refined visually by comparing predictions of ReefHab with reef locations known at that time [22]. Later, Kleypas et al. [28] determined new tolerance limits (K99) with the approach that we have adopted in our study. By using the WOA 2009, we obtain results more similar to K99 than K97 (Table 1). We also found that the Ω_ara_ threshold below which coral reefs disappear is 2.82, which contrasts with the value of 3.28 suggested earlier by Kleypas et al. [28].

**Table 1:** Tolerance limits for coral reefs associated to environmental variables.

K97 and K99					This study				
Variable	Source	Scale		Limits (Source)	Variable	Source	Scale		Limits
		Temporal	Spatial				Temporal	Spatial	
Temperature (°C)	Reynolds and Marsico, 1993	weekly	1° × 1°	18.1–31.5* (K97)	Temperature (°C)	Locarnini et al., 2010	annual	1° × 1°	**21.7–29.6**
				16.0–34.4 (K99)		NOAA OI SST V2	weekly		15.7–35.5
Salinity (psu)	Levitus, 1994	monthly	1° × 1°	30.0–39.0 (K97)	Salinity (psu)	Antonov et al., 2010	annual	1° × 1°	**28.7–40.4**
				23.3–41.8 (K99)			monthly		25.4–41.1
Nitrate (μmol L^-1^)	Levitus, 1993	monthly	1° × 1°	2.0** (K97)	Nitrate (μmol L^-1^)	Garcia et al., 2010	annual	1° × 1°	**4.51**
				3.34** (K99)					
Phosphate (μmol L^-1^)	Levitus, 1993	monthly	1° × 1°	0.2** (K97)	Phosphate (μmol L^-1^)	Garcia et al., 2010	annual	1° × 1°	**0.63**
				0.54** (K99)					
Topography (m)	Sloss, 1986	—	5’ × 5’	Z < Z_max_	Topography (m)	GEBCO_08 Grid	—	30”× 30”	Z < Z_max_
PAR (μmol photons m^-2^ s^-1^)	Pinker & Laszlo, 1992	monthly	2.5° × 2.5°	—	PAR (μmol photons m^-2^ s^-1^)	SeaWiFS Level 3	annual	5’ × 5’	—
K_490_ (m^-1^)	Arnone et al., 1992	monthly	0.16°× 0.16°	—	K_490_ (m^-1^)	SeaWiFS Level 3	annual	5’ × 5’	—
I_min_ (μmol photons m^-2^ s^-1^)	—	—	—	250–300	I_min_ (μmol photons m^-2^ s^-1^)	—	—	—	**450**
Ω_arag_	Archer, 1996	annual	2° × 2°	3.28 (K99)	Ω_arag_	Key et al., 2004	annual	1° × 1°	**2.82**

As explained in the main text, K97 refers to the limits suggested by Kleypas (1997) and K99 refers to the limits suggested by Kleypas et al. (1999). The new tolerance limits are highlighted in bold.

* 15.0 < T < 33.5 for enclosed seas.

** Annual average, as reported by the original study.

**Fig 3:**
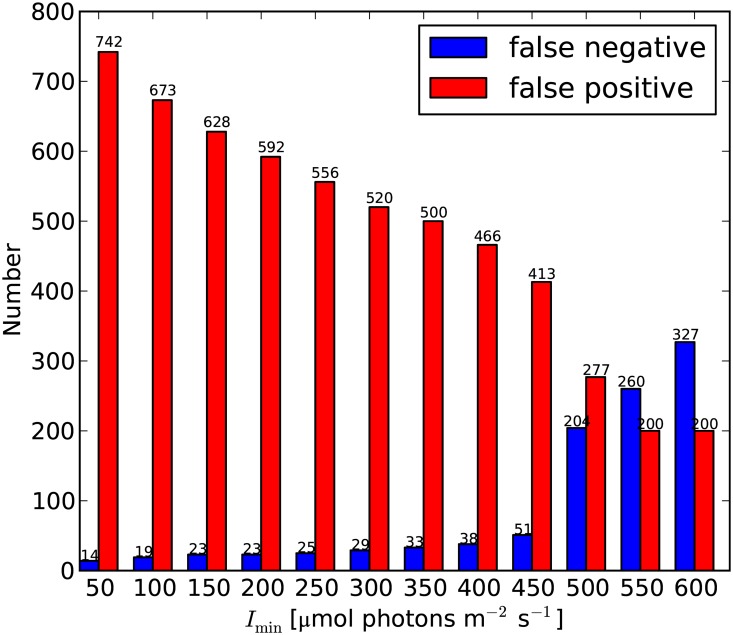
Number of false negatives and false positives obtained at different *I*_min_ values. False negatives and false positives steadily increase and decrease, respectively. A sharp shift in both false negatives and false positives is observed at *I*_min_ = 450 μmol photons m^-2^ s^-1^.

As mentioned in the Methods section, the new *I*_min_ was determined with a standard optimization technique. When *I*_min_ is increased from 50 to 450 μmol photons m^-2^ s^-1^, false positives decrease steadily from 742 to 413, whereas false negatives increase from 14 to 51 (Fig 3). From *I*_min_ = 450 μmol photons m^-2^ s^-1^ to *I*_min_ = 500 μmol photons m^-2^ s^-1^, false positives further decrease from 413 to 200, whereas false negatives increase rather abruptly from 51 to 327 (Fig 3). False positives do not indicate an erroneous result because the model estimates if reefs can “potentially” occur. In contrast, false negatives are to be avoided because they represent the case in which the model fails to predict a suitable habitat in a location where reefs do actually occur.

**Fig 4:**
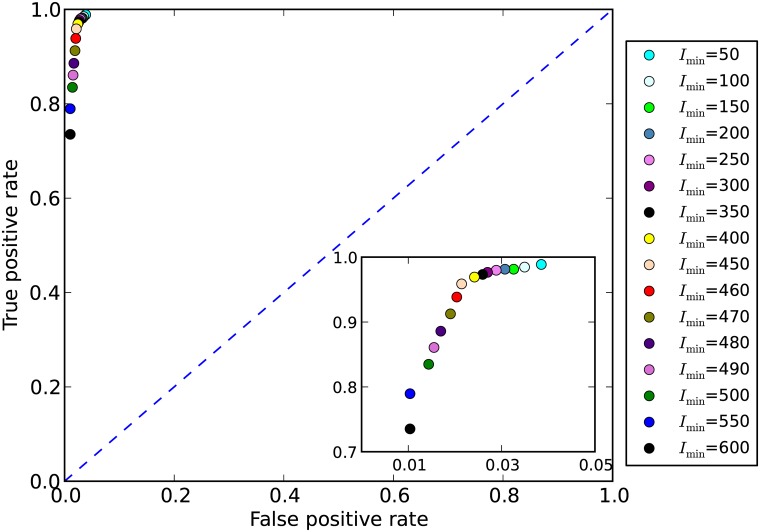
Receiver Operating Characteristic (ROC) graph. The graph shows the true positive rate versus the false positive rate. *I*_min_ = 450 μmol photons m^-2^ s^-1^ (pink dot) is the nearest to the (0, 1) point in the graph and therefore represents the best model result. The small inset is a zoom of the graph area where all the data points occur. The dashed blue line represents points in which true positive rates equal false positive rates.

The model response at different *I*_min_ was further analysed with the ROC graph (Fig 4). Due to the strong response in false positives and false negatives when *I*_min_ changes from 450 to 500 μmol photons m^-2^ s^-1^, we further investigated the model response in this *I*_min_ range with a finer step width of 10 μmol photons m^-2^ s^-1^. The best TPR to FPR ratio, i.e. the closest value to the perfect classification point (0,1) in the ROC graph, is obtained with *I*_min_ = 450 μmol photons m^-2^ s^-1^.

When ReefHab is run with the K97 tolerance limits, the model produces 473 false negatives. With our newly derived tolerance limits, false negatives are decreased to 51. Note that false negative model decisions could not be totally avoided due to the problems with bathymetry, as described in the (S1 Text and S1 Fig). The strong decrease in false negatives obtained with our tolerance limits is accompanied by a very minor increase in false positives, from 398 (K97 limits) to 413 (new limits), see S2 and S3 Figs.

### Potential reef habitats predicted by new environmental variables and K97 tolerance limits

**Fig 5:**
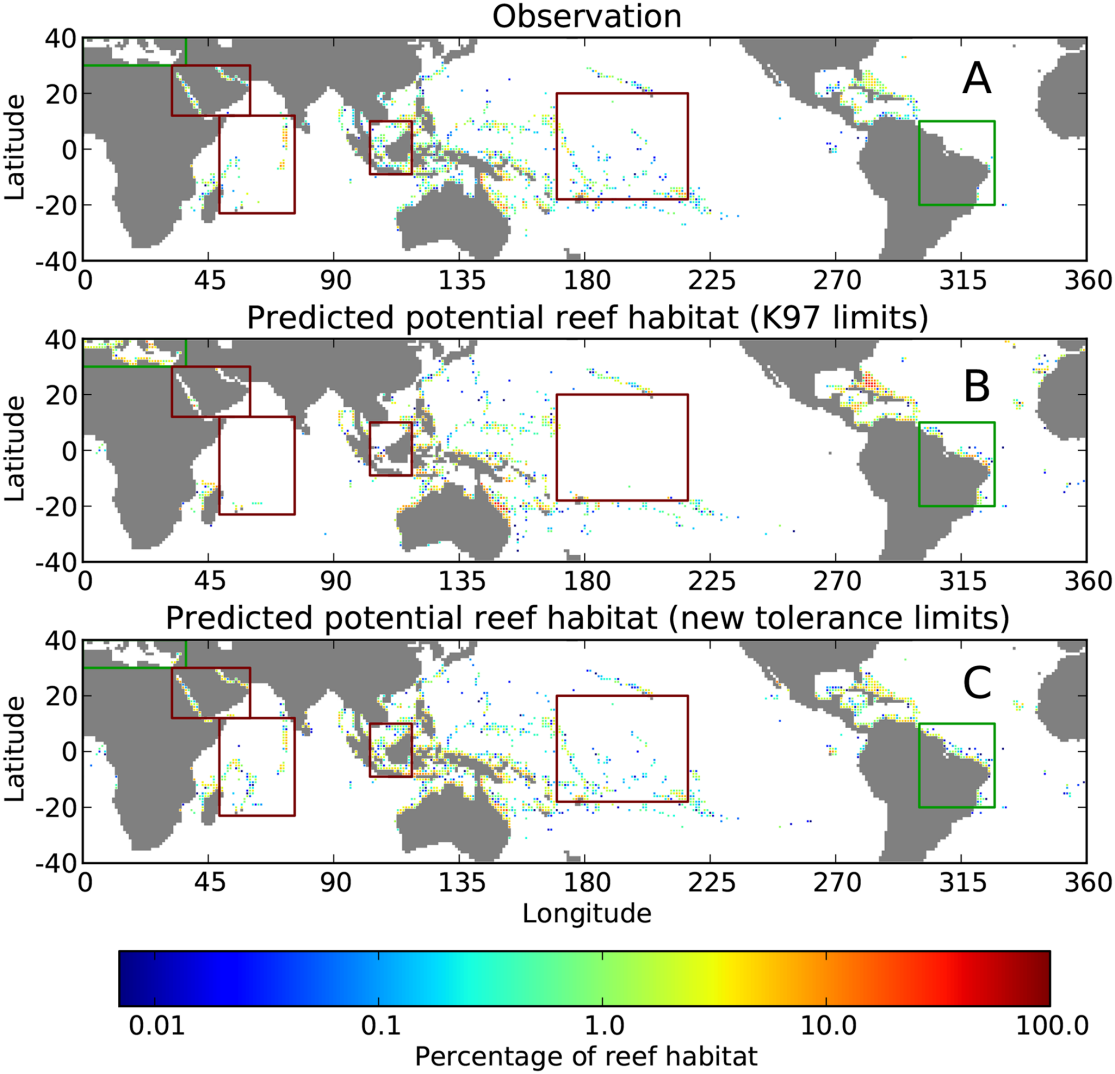
Observed coral reefs (A) and potential reef habitats predicted by ReefHab with the K97 tolerance limits (B) and with the tolerance limits derived in this study (C). All maps are presented on a 1° × 1° spatial resolution. Green rectangular boxes highlight areas where ReefHab overestimates the occurrences of potential reef habitats with respect to observations, whereas red boxes highlight areas where the occurrences of potential reef habitats are underestimated. The K97 tolerance limits fail to predict potential reef habitats in the Red Sea and Gulf of Aden, in the central Indian Ocean and central Pacific Ocean, and in the Indonesian Sea (B). These reefs are correctly captured by the new tolerance limits (C). The potential reef habitats predicted in the Mediterranean with the K97 limits are not produced with the new tolerance limits. Both tolerance limits predicted suitable potential reef habitats along the Brazilian coast, although the presence of reefs there is not confirmed by observations.

Fig 5A and 5B show a comparison between the predictions of potential reef habitats, obtained by running ReefHab with the most recent environmental variables (excluding Ω_ara_) in combination with the K97 tolerance limits, and the observed coral-reef distribution. The model reproduces a reasonable general pattern of potential reef habitats in the tropical and subtropical ocean although with some exceptions. For example, the model overestimates the occurrence of coral reefs in the Mediterranean Sea and it underestimates the occurrence of reefs in the Red Sea and in the Persian Gulf. The model does not capture the coral reefs of the Indian Ocean, Seychelles, Chagos Archipelago, and Maldives. In Southeast Asia, the model underestimates the occurrence of some coral reefs in the Java Sea and the Flores Sea and over-predicts reefs in the central and western coasts of northern Australia. In the Pacific, the model does not capture some small reefs such as Johnston Atoll, Palmyra Atoll, Tuvalu, Howland Island, and Galápagos Island. The model, however, performs well in the Atlantic/Caribbean region although the occurrence of reefs is overestimated along the Brazilian coasts.

### Potential reef habitats predicted by new environmental variables and new tolerance limits

The predicted potential reef habitats obtained with the tolerance limits derived in this study (Table 1 highlighted in bold) are consistent with the observed coral-reef distribution (Fig 5A and 5C). The model, correctly, does not predict the presence of coral reefs in the Mediterranean Sea, although it overestimates coral reefs in the Gulf of Oman and in the Gulf of Aden. Other places where the model overestimates coral reefs are the Seychelles, Mauritius, and the Andaman Sea. The model performs very well in Southeast Asia, along Australian coasts, in the Pacific Ocean, and in the Atlantic Ocean, especially in the western Pacific, where some small reefs (e.g. Johnston Atoll, Palmyra Atoll, and Tuvalu) that could not be captured with the K97 tolerance limits (see Fig 5 and S2 and S3 Figs) are now correctly predicted. Reef habitats along the Brazilian and northwestern Australian coasts are still somewhat overestimated (Fig 5C).

**Fig 6:**
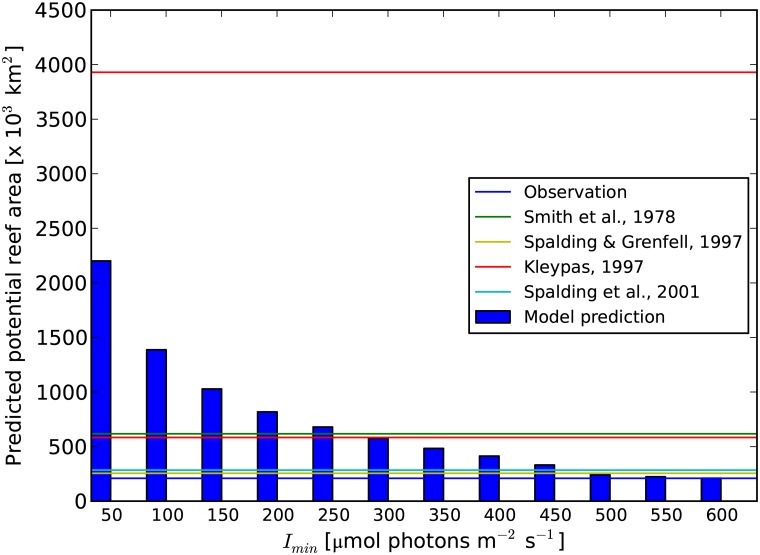
Area of potential reef habitats at different *I*_min_ values. The blue line represents the observed area covered by reefs; the green line represents the reef area estimated by Smith (1978), the yellow line represents the reef area estimated by Spalding & Grenfell (1997), the red line represents the potential reef habitat area estimated by Kleypas (1997), and the light blue line represents the potential reef habitat area estimated by Spalding et al. (2001).

With the tolerance limits derived in this study (Table 1), we estimate a global potential reef habitat area of about 330.5 × 10^3^ km^2^. The actual area where coral reefs are observed is about 209.5 × 10^3^ km^2^ (Fig 6).

## Discussion

### Tolerance limits for coral reefs

Temperature, salinity, nutrients, aragonite saturation state, and light are among the most important factors in controlling the geographic distribution of shallow-water coral reefs [17,28,41]. Global warming, ocean acidification, eutrophication, and other environmental perturbations can thus have negative consequences on corals by changing their habitats. Quantifying the suitable environmental ranges for coral reefs is a critical prerequisite for predicting the distribution of coral reefs in the future and for assessing the impacts that climate change may have on the reef ecosystem. Here we used the diagnostic model ReefHab [22] in combination with the most updated environmental data and high resolution bathymetry to derive potential reef habitats in the tropical and subtropical oceans. We found that the presence of several reefs is not predicted (e.g. Seychelles, and Maldives, and reefs in the Java Sea) when the model is forced with the K97 tolerance limits (see S2 Fig). This is because the K97 limits for nutrients, especially phosphate, are lower than the concentrations observed in those regions. We therefore derived the current environmental ranges suitable for coral reefs by running ReefHab in an inverse mode.

The K97 and K99 limits were provided on different temporal time scales (weekly, monthly and annually averaged). For comparison purposes, we computed the new environmental tolerance ranges on the same time scales although, as explained in the Methods section, our focus lies on the annually averaged conditions that sustain coral reefs. The newly derived limits for temperature are similar to the K97 and K99 limits when using weekly data (Table 1). On an annual basis, however, the temperature range resulting from our study (21.7–29.6°C) is narrower than that obtained with weekly data (15.7–35.5°C), because extreme values are smoothed out by the longer-term average. Short-term (from hours to weeks) laboratory and field studies have investigated the thermal tolerance for growth in common species of reef-building corals [14,42]. While exposure to extreme temperatures for a sufficiently long time induces bleaching [43,44], and can lead to massive coral mortalities [45–47], this occasional perturbation does not necessarily preclude the recovery of the ecosystem and the long-term suitability of the reef habitat [48]. Only if the frequency of such catastrophic events increases, the habitat may become unsuitable for corals, but this would be reflected in the long-term trend of the observed annual temperatures.

In contrast to the temperature, the tolerance range for salinity that we obtained on a monthly basis is similar to the K99 limits, whereas it is wider than the K97 limits. When calculated on an annual basis, the range becomes narrower than when using the monthly data, because again the values are smoothed by the longer-term average. The upper limit is determined by the Red Sea, which has the highest salinities (up to 41.1 psu) of all ocean waters. Whereas the lower limit is determined by the Gulf of Thailand, which experiences salinity values as low as 25.4 psu during the rainy season. Such broad tolerance limits for salinity are consistent with evidence suggesting that corals’ metabolic performance is only weakly sensitive to changes in this variable [18,42,49,50].

The annual thresholds for nitrate and phosphate that we obtained are up to three times higher than the K97 limits, but similar to the K99 limits (Table 1). These higher nutrient thresholds predicted by our study with respect to K97 are associated to the presence of coral reefs in areas adjacent to the Galápagos Islands and are, conceivably, due to deep-water upwelling in that region. When forced with the K97 limits, however, our model produced more false negatives than with our newly derived limits, especially with respect to phosphate. The new limits for nutrients improved the predictions of potential reef habitats in the Indonesian Sea, the central Pacific Ocean, the Seychelles, the Chagos Archipelago, and the Maldives, and they generated more false positives than the K97 limits in the Arabian Sea, the Bay of Bengal, the South China Sea, the central and eastern Pacific, and the Atlantic (see S2 and S3 Figs).

Kleypas [22] estimated the minimum light intensity necessary for coral reef habitats (*I*_min_) in the range of 250–300 μmol photons m^-2^ s^-1^ by comparing the total reef area predicted by ReefHab with the estimate of Smith in 1978 [51]. Newer estimates, however, suggest smaller areas for global coral-reef cover [4,52], which are also in accordance to our results (Fig 6). Our optimization procedure suggests that *I*_min_ = 450 μmol photons m^-2^ s^-1^ is a more plausible minimum light threshold for coral-reef habitat in today's ocean waters. This higher *I*_min_ value we found with respect to earlier works produces, consistently, a smaller total coral-reef area than the previous studies [22,51]. We also found a trade-off emerging between the accuracy in reef distribution patterns and the potential reef area predicted by the model when varying *I*_min_. Specifically, when *I*_min_ increases from 50 to 450 μmol photons m^-2^ s^-1^, the model predictions in terms of both coral-reef distribution patterns and habitat area become more accurate (i.e. less false negatives are produced). With *I*_min_ = 600 μmol photons m^-2^ day^-1^, the potential habitat area is closest to the observations (209.49 × 10^3^ km^2^ predicted vs. 209.68 × 10^3^ km^2^ observed), but such a good match is obtained at the cost of a less accurate prediction of reef distribution patterns (i.e. at the cost of increased false negatives). The minimum light intensity necessary for coral reefs that we found here reflects, therefore, the best balance between distribution patterns and reef area or, in other words, between (1) false predictions, i.e. false positives and false negatives (Fig 3), and (2) the correct outcomes, i.e. true positives and true negatives, as inferred from the ROC graph (Fig 4). Light penetration obviously varies with latitude and with the distance from shore. The *I*_min_ derived in the present study, however, represents a spatially averaged minimum light intensity for coral-reef habitats. Also different coral species can be characterised by different *I*_min_ values. *Pocillopora damicornis* from Hawaii, for example, has a higher *I*_min_ [53] than *Pavona praetorta* from the Marshall Islands [54]. And even within the same species, the minimum light tolerance can differ due to morphological reasons [53]. Our study, however, considers the reef community as a whole and the light tolerances reported for some corals [53,54], which are lower than what we found here, may not be representative of large natural environments supporting the development of very diverse reef communities. Note also that the *I*_min_ found here reflects the minimum irradiance levels required for coral-reef growth as averaged over a whole year. Obviously, the actual light conditions experienced in coral-reef waters can vary strongly on shorter time scales (from seasonal to daily).

Kleypas et al. [28] suggested a lower threshold for Ω_ara_ of 3.28. This limit has been adopted in the literature as “the standard value” below which no reefs occur [7,55]. Our study, however, suggests a lower threshold of 2.82 and shows that coral reefs in the Gulf of California, Galápagos, and northeast of Australia are found in waters where Ω_ara_ ranges between 2.82 and 3.28. The total reef area in these waters is 8.82 × 10^3^ km^2^, which accounts for about 4.2% of the global coral-reef coverage. Declining seawater pH due to the absorption of increasing atmospheric CO_2_, the process of ocean acidification, reduces carbonate ion concentrations and thus Ω_ara_ [7]. Several studies indicate that coral calcification decreases with declining Ω_ara_ [20,56–58]. In contrast, laboratory experiments show that some scleractinian coral species (e.g. *Oculina patagonica*) can survive acidified conditions (minimum pH = 7.3) for up to one year, although without accreting calcium carbonate [59]. Other species (*Stylophora pistillata*) can even calcify at Ω_ara_ values as low as 0.68 in the laboratory, albeit at rates lower than when subject to higher Ω_ara_ [60]. These environmental values, however, do not reflect present day ocean conditions. Consistently with our finding, field investigations in the natural environment suggest that coral reefs approach their natural limit at Ω_ara_ = 2.9 [61]. Unfortunately, the GLODAP dataset for DIC and TA does not cover the Indonesian Sea and the Caribbean. We can therefore not report on the aragonite saturation state in these areas. However, the lower threshold value of Ω_ara_ of 2.82 is found at the northern Great Barrier Reef, which is obviously covered by the GLODAP dataset.

De’ath et al. (2009) [62] showed that coral reefs of the northern Great Barrier Reef (GBR) are experiencing declining calcification rates since 1990 and suggested increasing temperature and declining Ω_ara_ as potential causes. Our results lend weight to their suggestion by showing that waters of the northern Great Barrier Reef are characterized by Ω_ara_ values close to the minimum threshold.

The optimization of the environmental boundary limits for coral-reef habitats helped us to substantially reduce the number of false negatives (i.e. to reduce the number of known coral-reef sites excluded by the model predictions) to only 51 occurrences (Fig 3 and S4 Fig). Therefore, despite its simplicity, ReefHab predicted the spatial distribution of potential reef habitats with good accuracy as compared to patterns of actual coral-reef occurrences. An earlier study compared the performances of other three different models in predicting the presence of coral reefs in shallow tropical waters [17]. Despite the higher complexities of these models with respect to ReefHab, they tend to produce a higher number of false negatives than ReefHab (for example compare Fig. 4 in ref. 17 with our S4 Fig).

### Potential reef habitat area

A precise estimate of the global coral reef habitat area is important for understanding the potential impact of changing environmental conditions on coral-reef biogeography. Different estimates of global coral-reef coverage are found in the literature [4,13,22,51,52,63], ranging from 250 × 10^3^ km^2^ [13] to 1500 × 10^3^ km^2^ [63]. The coral- reef area we determined from the newest charted reef data is about 209.5 × 10^3^ km^2^. Whether this lower value with respect to earlier estimates is an indication of a global decline in coral-reef cover or an improvement with respect to rather optimistic estimates is not easy to judge.

Our model results suggest a potential reef habitat of about 330.5 × 10^3^ km^2^. The model, however, predicts the “potential” reef habitat, which is by definition an overestimation of the “real” coral-reef area. In addition, the ReefHab model predicts potential reef habitats only as a function of six physical and chemical environmental factors: 1) temperature, 2) salinity, 3) nitrate, 4) phosphate, 5) aragonite saturation state, and 6) light. Besides these environmental factors, the world’s coral reefs also face threats from a wide range of human activities, including coastal development, runoff of fertilizer from agricultural activities, physical damages from anchors and ship groundings, overfishing, and tourism. Omitting these difficult to quantify factors may also lead to an overestimation of potential reef habitats with respect to the actual observations. Uncertainties can also affect the actual reef observations. For example, although in some regions the presence of reefs is well known (e.g. in Cape Verde [64], Gulf of Guinea [65], and our model correctly predicts their presence, these reefs have not yet been charted and therefore do not appear in the observational data. Additionally, new reefs are constantly being discovered [66].

In summary, by using the diagnostic ReefHab model, we were able to predict the global distribution of potential coral-reef habitats based on a number of physical and chemical variables, which then allowed us to determine annually and spatially averaged tolerance limits for coral reefs under current ocean conditions. New tolerance limits and the quantified potential reef habitats can allow us to predict the global reef distribution in the future under a changing climate. The potential coral-reef habitat area calculated with ReefHab is about 121 × 10^3^ km^2^ larger than the charted reefs. This indicates that the growth potential of coral reefs could be higher than currently observed in the absence of other anthropogenic perturbations such as fishing, local damage, and pollution.

## Supporting Information

S1 Fig Coral reef distribution (orange) around Moorea Island (17.53°N, 149.83°W) and Tahiti Island (17.67°N, 149.42°W) overlaied on the GEBCO_08 Grid bathymetry. Note the portions of reefs erroneously lying over very deep waters (dark blue spots).
(TIF)

S2 Fig The distribution of false positives (left panels) and false negatives (right panels) obtained by running ReefHab with the K97 limits and with *I_min_* = 300 μmol photons m^-2^ s^-1^ for each environmental variable. The maps are on a 1° × 1° spatial resolution. Only considering temperature, given the definition of false positive, the red grid cell in false positive for temperature represents the area within the tolerance limits we set for potential reef habitats, but no observed reef found in that area. False positives contribute to overestimation of reef areas, for example, in Mediterranean Sea where non-reef corals there. The same way of interpreting the information for false negative, red grid cells contribute underestimation of observed reef, for example, due to unsuitable phosphate tolerance, lots of reefs are not captured by ReefHab in Indo-Pacific region.
(TIF)

S3 Fig False positives (left panels) and false negatives (right panels) obtained by running ReefHab with the new environmental tolerance limits (this study) and with *I_min_* = 450 μmol photons m^-2^ s^-1^. The same way of interpreting the information as in S2 Fig Noticeably, the GLODAP dataset for DIC and TA does not cover the Indonesian Sea and the Caribbean. We created a mask for these two regions, when ReefHab checks these two regions, only temperature, salinity, nitrate, phosphate, and light condition are considered.
(TIF)

S4 Fig Total 51 false positives obtained by running ReefHab combined with all the new environmental tolerance limits (this study) and with *I_min_* = 450 μmol photons m^-2^ s^-1^. (TIF)

S1 Text Mismatch between bottom topography and coral reef data. (DOCX)
