# Supplementary figures and images for: Suitable Environmental Ranges for Potential Coral Reef Habitats in the Tropical Ocean
Source: PLoS One. 2015 Jun 1;10(6):e0128831. doi: 10.1371/journal.pone.0128831 (PMC4452591; doi:10.1371/journal.pone.0128831)

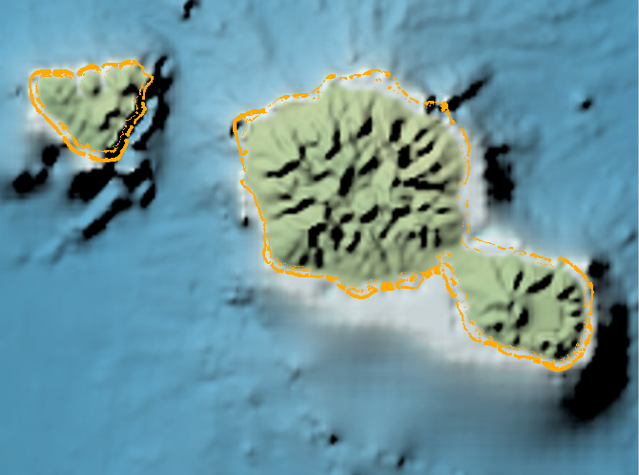

Supplement: S1 Fig — Note the portions of reefs erroneously lying over very deep waters (dark blue spots). (TIF) [file pone.0128831.s001.tif]

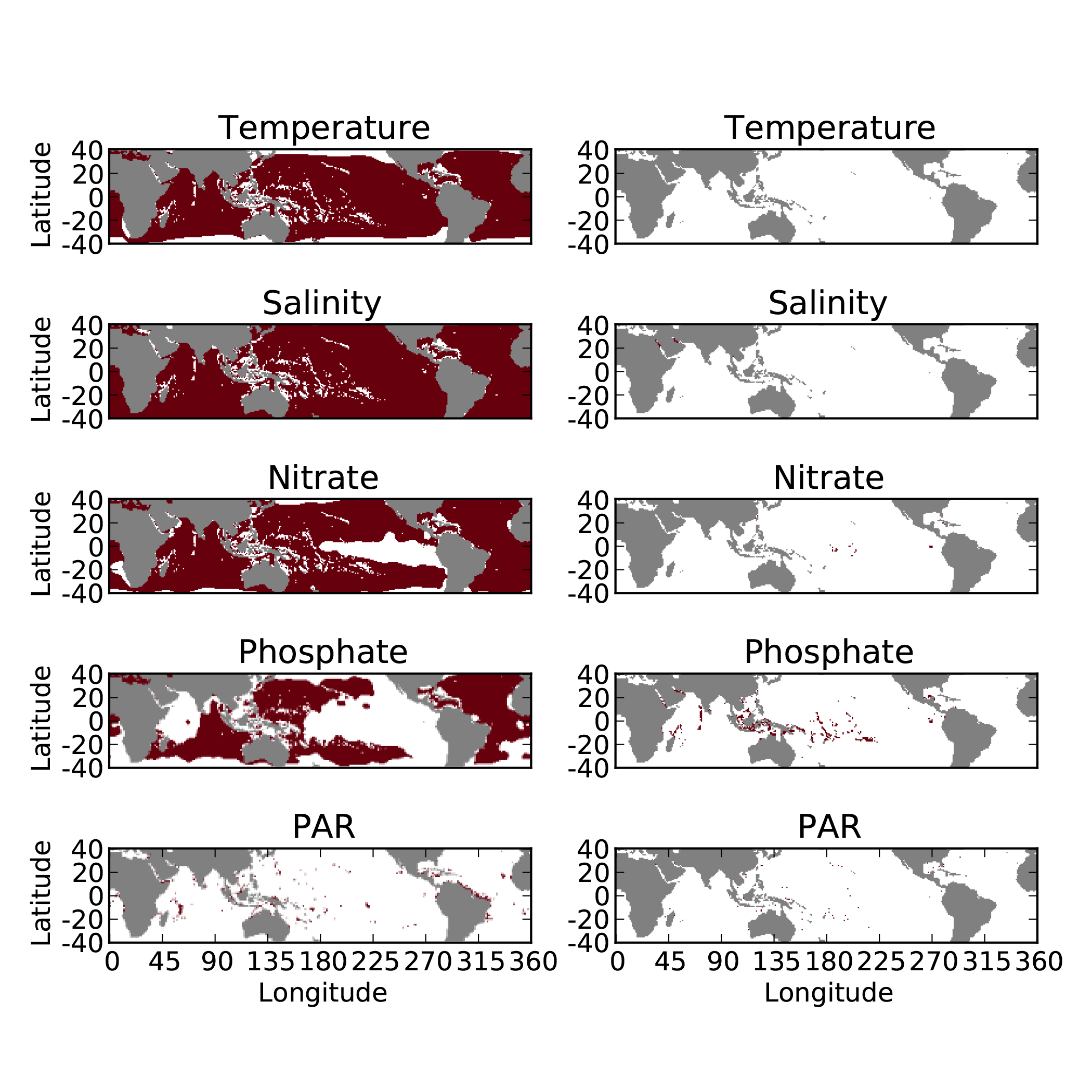

Supplement: S2 Fig — The maps are on a 1° × 1° spatial resolution. Only considering temperature, given the definition of false positive, the red grid cell in false positive for temperature represents the area within the tolerance limits we set for potential reef habitats, but no observed reef found in that area. False positives contribute to overestimation of reef areas, for example, in Mediterranean Sea where non-reef corals there. The same way of interpreting the information for false negative, red grid cells contribute underestimation of observed reef, for example, due to unsuitable phosphate tolerance, lots of reefs are not captured by ReefHab in Indo-Pacific region. (TIF) [file pone.0128831.s002.tif]

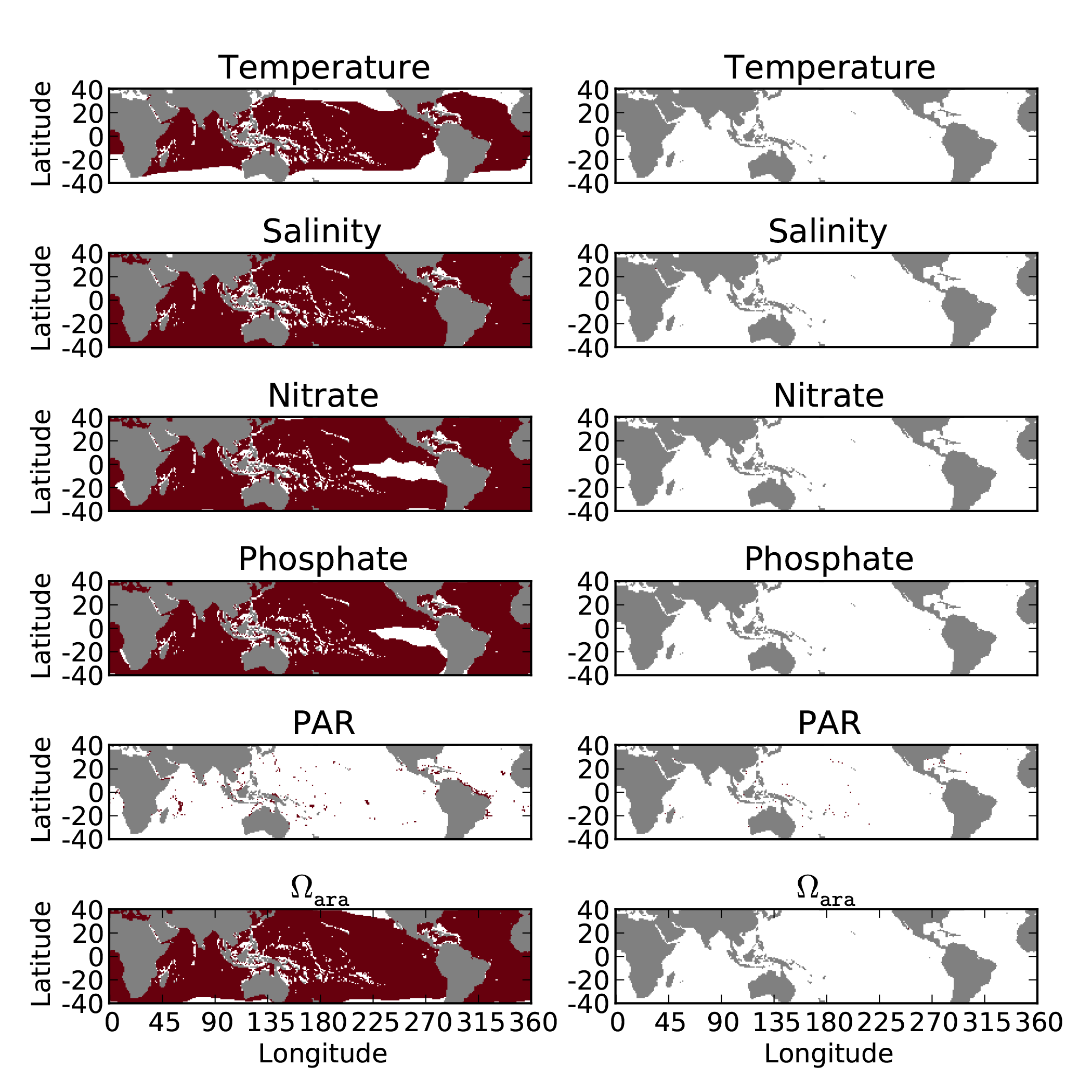

Supplement: S3 Fig — The same way of interpreting the information as in S2 Fig Noticeably, the GLODAP dataset for DIC and TA does not cover the Indonesian Sea and the Caribbean. We created a mask for these two regions, when ReefHab checks these two regions, only temperature, salinity, nitrate, phosphate, and light condition are considered. (TIF) [file pone.0128831.s003.tif]

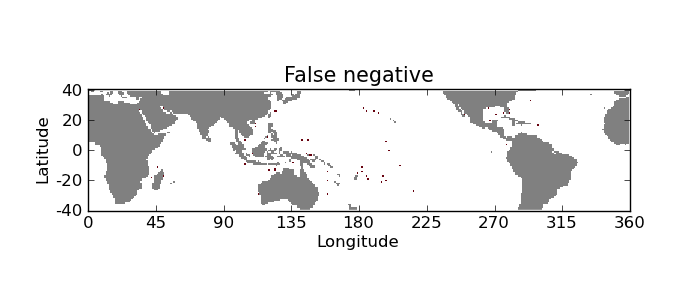

Supplement: S4 Fig — (TIF) [file pone.0128831.s004.tif]
