# Supplementary material for: Suitable Environmental Ranges for Potential Coral Reef Habitats in the Tropical Ocean
Source: PLoS One. 2015 Jun 1;10(6):e0128831. doi: 10.1371/journal.pone.0128831 (PMC4452591; doi:10.1371/journal.pone.0128831)
Supplement: S1 Text — (DOCX) [file pone.0128831.s005.docx]

**Mismatch between bottom topography and coral reef data**

The projection of the coral reef distribution data onto the most recent bottom topography reveals an important discrepancy between the bathymetry data (GEBCO_08) and the charted coral reef locations the Global Distribution of Coral Reefs (2010) (IMaRS-USF & IRD, 2005; IMaRS-USF, 2005; UNEP-WCMC, WorldFish Centre, WRI and TNC, 2010). The depth at the charted coral reef locations is frequently found to be deeper than 2000 m and up to 7000 m, which is obviously incorrect for tropical warm water corals. As an example, Moorea Island is a high island in French Polynesia, 17 km northwest of Tahiti, located in the archipelago of the Society Islands in the southern Pacific Ocean. The coral reefs at the eastern shore of the small island correspond to very deep waters (see S1 Fig).

A similar mismatch occurs in many atolls of French Polynesia and at any location where the GEBCO_08 bathymetry results, despites its high resolution of 30” x 30”, rather coarse.

The GEBCO_08 Grid with 30 arc-second resolution is up to date the most accurate bottom topography available and much more accurate than ETOPO1 or ETOPO5 with 1 arc-minute and 5 arc-minute resolutions, respectively. However, the GEBCO_08 bathymetry is created from a combination of ship based echo sounder data and satellite derived gravimetric data, wherever no ship data are available, which is often the case in remote and scarcely populated ocean regions like Oceania. An ocean basemap reconstructed on the basis of satellite gravimetric data will never be so accurate as to resolve coral reef structures and more ship based measurements are required to refine available topography datasets. In addition, errors may also affect the bathymetry data, like islands in the dataset that simply do not exist in reality (Seton et al., 2013).

Given these uncertainties in the available bathymetry data we performed a sensitivity analysis for different minimum light intensities to estimate the most suitable light level for coral growth, as described in the main text.

**Reference**

Seton, M., Williams, S., Zahirovic, S., & Micklethwaite, S. (2013). Obituary : Sandy Island (1876-2012). *Eos, Transactions American Geophysical Union*, *94*(15), 1–3.
